# Correcting cardiorespiratory noise in resting-state functional MRI data acquired in critically ill patients

**DOI:** 10.1093/braincomms/fcac280

**Published:** 2022-10-31

**Authors:** Suk-Tak Chan, William R Sanders, David Fischer, John E Kirsch, Vitaly Napadow, Yelena G Bodien, Brian L Edlow

**Affiliations:** Athinoula A. Martinos Center for Biomedical Imaging, Department of Radiology, Massachusetts General Hospital and Harvard Medical School, Charlestown, MA 02129, USA; Center for Neurotechnology and Neurorecovery, Department of Neurology, Massachusetts General Hospital and Harvard Medical School, Boston, MA 02114, USA; Center for Neurotechnology and Neurorecovery, Department of Neurology, Massachusetts General Hospital and Harvard Medical School, Boston, MA 02114, USA; Athinoula A. Martinos Center for Biomedical Imaging, Department of Radiology, Massachusetts General Hospital and Harvard Medical School, Charlestown, MA 02129, USA; Athinoula A. Martinos Center for Biomedical Imaging, Department of Radiology, Massachusetts General Hospital and Harvard Medical School, Charlestown, MA 02129, USA; Department of Physical Medicine and Rehabilitation, Spaulding Rehabilitation Hospital, Harvard Medical School, Boston, MA 02129, USA; Center for Neurotechnology and Neurorecovery, Department of Neurology, Massachusetts General Hospital and Harvard Medical School, Boston, MA 02114, USA; Department of Physical Medicine and Rehabilitation, Spaulding Rehabilitation Hospital, Harvard Medical School, Boston, MA 02129, USA; Athinoula A. Martinos Center for Biomedical Imaging, Department of Radiology, Massachusetts General Hospital and Harvard Medical School, Charlestown, MA 02129, USA; Center for Neurotechnology and Neurorecovery, Department of Neurology, Massachusetts General Hospital and Harvard Medical School, Boston, MA 02114, USA

**Keywords:** physiological noise, functional MRI, brain network, connectivity, traumatic brain injury

## Abstract

Resting-state functional MRI is being used to develop diagnostic, prognostic and therapeutic biomarkers for critically ill patients with severe brain injuries. In studies of healthy volunteers and non-critically ill patients, prospective cardiorespiratory data are routinely collected to remove non-neuronal fluctuations in the resting-state functional MRI signal during analysis. However, the feasibility and utility of collecting cardiorespiratory data in critically ill patients on a clinical MRI scanner are unknown. We concurrently acquired resting-state functional MRI (repetition time = 1250 ms) and cardiac and respiratory data in 23 critically ill patients with acute severe traumatic brain injury and in 12 healthy control subjects. We compared the functional connectivity results from two approaches that are commonly used to correct cardiorespiratory noise: (i) denoising with cardiorespiratory data (i.e. image-based method for retrospective correction of physiological motion effects in functional MRI) and (ii) standard bandpass filtering. Resting-state functional MRI data in 7 patients could not be analysed due to imaging artefacts. In 6 of the remaining 16 patients (37.5%), cardiorespiratory data were either incomplete or corrupted. In patients (*n* = 10) and control subjects (*n* = 10), the functional connectivity results corrected with the image-based method for retrospective correction of physiological motion effects in functional MRI did not significantly differ from those corrected with bandpass filtering of 0.008–0.125 Hz. Collectively, these findings suggest that, in critically ill patients with severe traumatic brain injury, there is limited feasibility and utility to denoising the resting-state functional MRI signal with prospectively acquired cardiorespiratory data.

## Teaser

Chan *et al*. reported a limited feasibility and utility to prospectively acquire high-quality cardiorespiratory data during resting-state functional MRI in critically ill patients with severe traumatic brain injury for physiological correction.

## Graphical Abstract

**Graphical Abstract:**
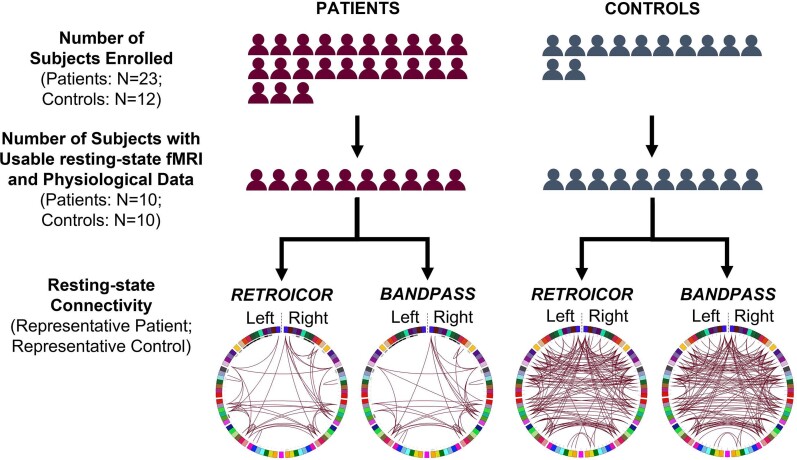

## Introduction

Over the past decade, resting-state functional MRI (rs-fMRI) studies have demonstrated the potential diagnostic and prognostic utility of brain network mapping in patients with a broad range of brain injuries, including traumatic brain injury (TBI),^1–4^ hypoxic–ischaemic injury from cardiac arrest^2,5–8^ and hypoxic injury from coronavirus disease 2019.^9,10^ In addition, rs-fMRI is now used as a pharmacodynamic biomarker in early-stage clinical trials to assess brain responses to targeted therapies aimed at promoting recovery of consciousness.^11^ Nevertheless, despite the growing evidence that rs-fMRI has potential as a diagnostic, prognostic and therapeutic biomarker, the optimal data acquisition, pre-processing and analysis methods for rs-fMRI have not been determined for critically ill patients with acute brain injuries. For example, a fundamental pre-processing question is how to account for non-neuronal, physiological fluctuations, such as cardiac pulsation and respiration. These cardiorespiratory signal fluctuations contribute noise to the data, obscuring the blood oxygenation level dependent (BOLD) signals used to identify functional network connectivity. Accounting for these cardiorespiratory signal fluctuations during pre-processing is particularly relevant in critically ill patients, who may experience fluctuations in vital signs during the rs-fMRI scan, causing potentially unpredictable changes in the BOLD signal. As a result, the underlying neuronal dynamics measured by functional network connectivity may be masked.

Standard bandpass filtering has been used in the early rs-fMRI studies to reduce the noise induced by cardiorespiratory fluctuations.^12–14^ However, later studies in healthy human subjects have shown that, compared with standard bandpass filtering, prospective acquisition of cardiorespiratory data may improve the signal-to-noise properties of spontaneous BOLD fluctuations,^15–18^ because these cardiorespiratory data facilitate signal correction based on subjects’ unique cardiac and respiratory oscillations. In addition, cardiac pulsation and respiration signals have different frequencies and amplitude characteristics^19^ and therefore may not be amenable to correction with a single bandpass filter. Notably, most studies that prospectively collected cardiorespiratory data enrolled healthy control subjects who had relatively stable haemodynamics, are able to tolerate long scan times and were scanned in research settings with precise instrumentation.^20,21^ Conversely, setting up additional equipment and optimizing the cardiorespiratory signals prolong scan time posing safety risks for critically ill patients. Patients in the intensive care unit (ICU) must be scanned on a clinical scanner using standard physiological monitoring instrumentation integrated with the MRI system, where the cardiorespiratory data acquired may be suboptimal for rs-fMRI signal denoising. Moreover, the quality of cardiorespiratory data also depends on a patient’s injury burden, cardiopulmonary stability, haemodynamic fluctuations and cooperation. Involuntary motion is common in critically ill patients with altered consciousness,^3,22,23^ and the placement of physiological sensors can be complicated by concurrent injuries to the chest, abdomen and extremities. Indeed, these safety, scientific and clinical challenges likely explain why few fMRI studies have been performed on critically ill patients^23^ and why there have been no studies, to our knowledge, acquiring cardiorespiratory data specifically for the pre-processing of rs-fMRI data in critically ill patients.

There are multiple advantages and limitations of pre-processing rs-fMRI using the prospective acquisition of cardiorespiratory data and standard bandpass filtering. In this prospective, observational study, we investigated the feasibility of acquiring cardiorespiratory data during rs-fMRI in critically ill patients with acute severe TBI and the utility of using these data to correct for non-neuronal signals in the BOLD data. The study was performed on a 3 T MRI scanner located in the Neurosciences ICU (NeuroICU) at an academic medical centre. We hypothesized that cardiorespiratory data acquisition is feasible in a clinical setting for critically ill patients and that it reduces physiological confounds more effectively than does bandpass filtering. In the feasibility analysis, we assessed the success rate of acquiring technically useful, uncorrupted cardiac and respiratory data. In the utility analysis, we compared the functional connectivity brain maps corrected with image-based method for retrospective correction of physiological motion effects in fMRI (RETROICOR)^15^ to those corrected with standard bandpass filters. We selected these two approaches for comparison because they are automated and frequently used in rs-fMRI studies.^3,15–18,24–27^ Recognizing that there is no gold-standard for ‘ground-truth’ physiological correction of functional brain connectivity, we tested the hypothesis that RETROICOR correction using cardiorespiratory signals yields higher levels of network connectivity than does standard bandpass filtering. Our goal is to use these results to inform the design of future studies that require physiological correction of rs-fMRI data for mapping brain connectivity in critically ill patients.

## Materials and methods

### Participants

We enrolled 12 healthy volunteers and 23 patients with acute severe TBI, defined by a post-resuscitation Glasgow Coma Scale score ≤ 8 prior to admission to an ICU. All the patients were enrolled consecutively from the NeuroICU, surgical ICU, or multi-disciplinary ICU at Massachusetts General Hospital (MGH), as part of an ongoing observational study (ClinicalTrials.gov NCT03504709). Healthy control subjects had no history of brain injuries, neurological disease, psychiatric disease, cardiovascular disease, or any history of diabetes, hypertension, or renal disease and were recruited by e-mail and poster placement within the MGH hospital network.

All components of this study were performed in compliance with the Declaration of Helsinki, and all procedures were approved by the hospital’s Human Research Committee. Written informed consent was obtained from healthy subjects and patients’ surrogate decision-makers.

### MRI acquisition

We performed the MRI on a 3 T Skyra scanner (Siemens Medical, Erlangen, Germany) in the NeuroICU with a 32-channel head coil. Foam pads and inflatable positioning pads were used to minimize head motion. We acquired the following MRI datasets on each subject: (i) high-resolution sagittal images acquired with volumetric T1-weighted three-dimensional multi-echo magnetization-prepared 180 degrees radio-frequency pulses and rapid gradient-echo sequence [TR (repetition time) = 2530 ms, TE (echo time) = 1.69 ms/3.55 ms/5.41 ms/7.27 ms, flip angle = 7°, FOV = 256 × 256 mm, matrix = 256 × 256, slice thickness = 1 mm] and (ii) BOLD-fMRI images acquired with echo-planar imaging sequence (TR = 1250 ms, TE = 30 ms, flip angle = 65°, FOV = 212 × 212 mm, matrix = 106 × 106, slice thickness = 2 mm, slice gap = 0 mm, duration = 10 min) while the subject was at rest. Subjects were instructed to keep their eyes open during the rs-fMRI scans. We aimed to acquire two rs-fMRI datasets for each subject—one at the beginning of the MRI scan, and one at the end of the scan—separated by ∼30 min of stimulus-based and task-based fMRI paradigms.

We optimized the MRI sequence with a short TR of 1.25 s and a resting-state scan length of 10 min. This made the maximum sampling frequency 0.4 hertz (Hz), allowing the removal of most of the fluctuations due to respiratory cycles. The narrow frequency spacing of about 0.002 Hz based on the scan duration (i.e. frequency spacing = 1/duration of scan in seconds; duration of scan = 1.25 s of TR × 482 time points = 602.5 s) offered proper sampling of signal changes down to 0.002 Hz and more precise removal of fluctuations when bandpass filtering strategy was applied.

We recorded the time series of both optical plethysmography and respiration using the Siemens Physiological Monitoring Unit (Siemens Healthcare, Erlangen, Germany). Optical plethysmography was measured instead of electrocardiogram for cardiac pulsation because the optical signals were less contaminated by the imaging gradient changes. Breath-by-breath respiratory cycles were measured by pressure changes in the pneumatic bladder located between the skin surface and the respiratory belt around the upper abdomen. All cardiorespiratory data recordings were synchronized using the timestamps in the data recordings and image headers.

### Data analysis

The imaging and cardiorespiratory data from the first rs-fMRI scan for each subject were used in the following analyses, except for one patient (P3) because the first rs-fMRI scan was terminated in the middle due to a technical issue with the head coil resulting in signal void in the anterior part of the brain. For this patient, the imaging and cardiorespiratory data from the second rs-fMRI scan were used.

#### Processing of cardiorespiratory data

We used Matlab R2020a (Mathworks, Inc., Natick, MA, USA) to analyse the cardiorespiratory data. The peaks on the optical plethysmography time series served as a surrogate of R peaks in electrocardiogram, while the peaks and troughs on the respiratory time series indicated end inspiration and end-expiration, respectively (Supplementary Fig. 1). Peaks and troughs on the time series of optical plethysmography and respiration were determined using a custom Matlab function^21^ and corrected on the graphical user interface. The cardiac phase used in RETROICOR^15^ advances linearly from 0 to 2π during each R-R interval and is reset to 0 for the next cycle. The inspiratory phase spans from 0 to π and the expiratory phase spans from 0 to −π.

#### Pre-processing of resting-state BOLD-fMRI data

**Figure 1:**
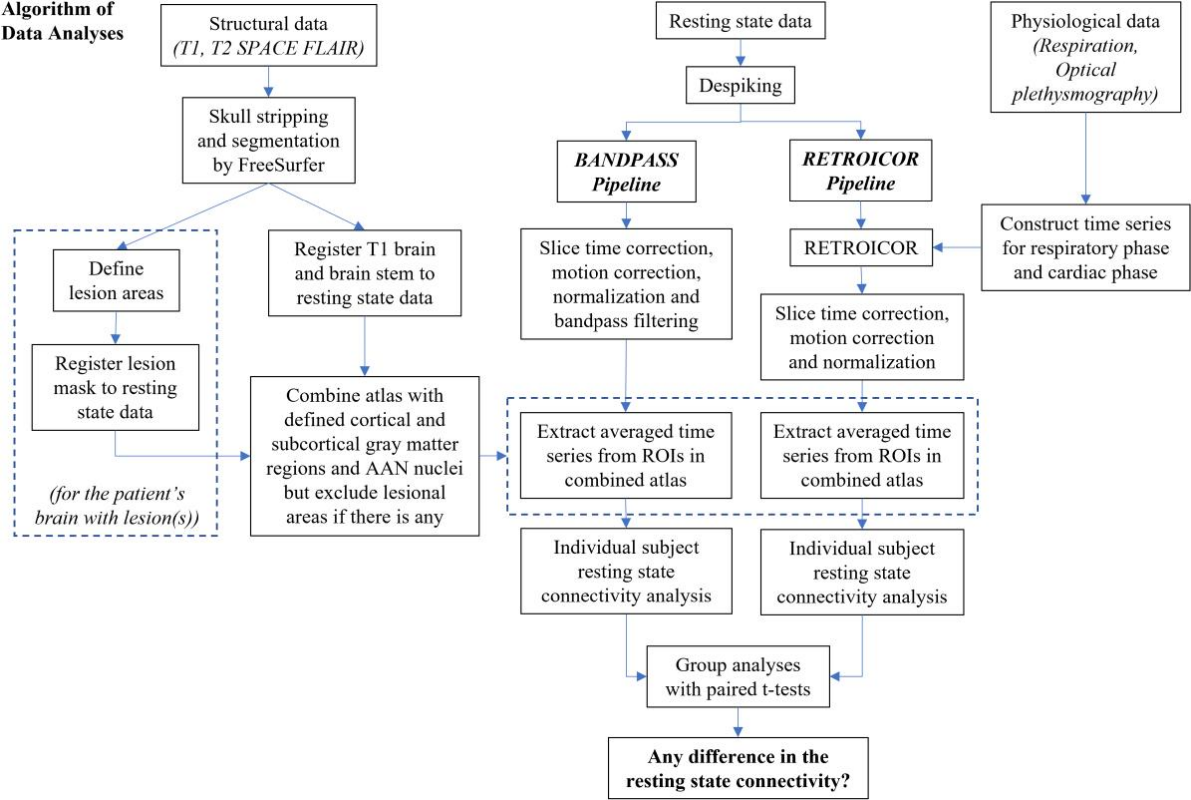
**Algorithm of data analyses**.

All BOLD-fMRI data were imported into the Analysis of Functional NeuroImage (AFNI) software^28^ (National Institute of Mental Health, http://afni.nimh.nih.gov) for pre-processing. The first 12 volumes of each functional dataset, collected before equilibrium magnetization was reached, were discarded. Artefactual spikes were removed from the time series in each voxel using ‘3dDespike’ in AFNI. In order to compare resting-state connectivity after RETROICOR versus standard bandpass filtering, the resting-state BOLD data were separately processed with two processing pipelines: RETROICOR (RETROICOR pipeline) and standard bandpass filtering (BANDPASS pipeline). We focused on RETROICOR, rather than other physiological correction methods that employ a component-based approach (e.g. CompCor), because some of these methods require accurate specification of a ‘noise’ region-of-interest in white matter or cerebrospinal fluid.^29^ In patients with distorted brain anatomy and BOLD signal changes in white matter due to traumatic microbleeds,^30^ defining a precise ‘noise’ region-of-interest is especially challenging. A schematic diagram of our data analysis algorithm with the two independent processing pipelines (RETROICOR and BANDPASS) is shown in Fig. 1.

#### RETROICOR pipeline

Time series of cardiac and respiratory phases were imported into the program ‘3dretroicor’ in AFNI to remove cardiac and respiratory motions of the resting-state BOLD-fMRI after despiking. Each functional dataset in the RETROICOR pipeline was corrected for slice timing, motion-corrected and co-registered to the first image of the functional dataset using three-dimensional volume registration. Voxels located within the ventricles and outside the brain were defined in brain parcellation using FreeSurfer^31,32^ (MGH/MIT/HMS Athinoula A. Martinos Center for Biomedical Imaging, Boston, USA http://surfer.nmr.mgh.harvard.edu) and were excluded from subsequent analyses. In the co-registered functional dataset, motion outliers were defined as any timepoint with 5% of brain voxels having the averaged derivative change of translational and rotational motion parameters of more than 0.4. The co-registered dataset was then normalized to its mean intensity value across the time series for the per cent BOLD signal changes (ΔBOLD). In the normalized functional dataset, the time series of each voxel was detrended with the fifth order of polynomials to remove the low drift frequency (<0.005 Hz). The low drift frequency and the components of motion were removed in one single process using orthogonal projection. Individual subject brain volumes with time series of ΔBOLD were used in the connectivity analysis.

#### BANDPASS pipeline

Slice timing correction, motion correction, co-registration, normalization and removal of low drift frequency and motion components were applied to each functional dataset after despiking in the BANDPASS pipeline using the same approach as in the RETROICOR pipeline. Bandpass filters of different frequency bandwidths were separately applied to the clean functional dataset. The starting frequency of the bandpass filters was 2*^x^*, where *x* ranged from −7 (i.e. 2^−7^ = 0.008 Hz) to −3 (i.e. 2^−3^ = 0.125 Hz) at an increment of the exponential of +1. The ending frequency of the bandpass filters was 2*^y^*, where *y* ranged from −6 (i.e. 2^−6^ = 0.016 Hz) to −2 (i.e. 2^−2^ = 0.25 Hz) at an increment of the exponential of +1. A total of 15 frequency bandwidths were used as bandpass filters, and 15 filtered data sets were obtained.

#### Individual subject resting-state connectivity analysis

For each subject, we constructed a brain mask with 82 cortical and subcortical grey matter brain regions parcellated by the software FreeSurfer^31,32^ and 16 brainstem nuclei from the Harvard ascending arousal network (AAN) atlas.^33^ No single voxel was included in more than one brain region. If a subject had a large brain lesion, such as a haemorrhagic contusion, visible on the T1-weighted structural images, the study investigator (S.T.C.) constructed a lesion mask by manually drawing the lesion on the high-resolution T1-weighted images as a region-of-interest using FreeSurfer (http://surfer.nmr.mgh.harvard.edu), as done previously.^34^ The brain atlas with defined cortical and subcortical grey matter regions and AAN nuclei and the brain lesion masks were registered to brain volumes with time series of ΔBOLD via the first brain volume in the motion-corrected functional dataset. Brain regions that overlapped with brain lesion masks were excluded from the connectivity analysis.

For the normalized dataset in the RETROICOR pipeline, the averaged time series of ΔBOLD in each brain region were correlated with that of each other brain region using the program ‘3dNetCorr’.^35^ Pearson’s correlation coefficients were calculated from 3403 region pairs. In the BANDPASS pipeline, the same correlation analysis was applied to each normalized dataset. For both pipelines, the Pearson’s correlation coefficient from each region pair was transformed to a Fisher’s *z*-value, to indicate the connectivity strength for the group analysis.

#### Statistical analysis

Age as a continuous variable was summarized as median and interquartile range (IQR), and group (TBI and control) difference was assessed using the Kruskal–Wallis test. Sex as a categorical variable was summarized as frequencies; group difference was assessed using the *χ*^2^ test.

The connectivity strength indicated by Fisher’s *z*-values from patient and control groups were analysed separately. The potential difference in the brain connectivity for each region pair was first explored using paired *t*-test to compare the Fisher’s *z*-values derived from the RETROICOR pipeline and those from the commonly used bandpass filter of 0.008–0.125 Hz in the BANDPASS pipeline. Such a comparison was repeated for 3403 region pairs. False discovery rate was used to correct the multiple comparisons. A significant difference was considered at *P*_fdr_ < 0.05.

To further explore if the frequency bandwidth of 0.008–0.125 Hz was optimal for bandpass filtering of rs-fMRI data, we used Pearson’s correlation to measure the correlation of averaged connectivity strength between the RETROICOR pipeline and 15 bandpass filters in the BANDPASS pipelines. For each region pair, the mean Fisher’s *z*-value was calculated separately from the RETROICOR pipeline and each bandpass filter in the BANDPASS pipeline in the same group of subjects. The mean Fisher’s *z*-values from 3403 region pairs in two processing arms were correlated. A significant correlation was considered at *P* < 0.05.

Intraclass correlation was used to study the similarity of the connectivity strength between the two pipelines in each region pair. The intraclass correlation coefficients between the two pipelines for each region pair were calculated between the Fisher’s *z*-values from the RETROICOR pipeline and each bandpass filter in the BANDPASS pipeline in the same group of subjects. False discovery rate was used to correct for multiple comparisons. A significant correlation was considered at *P*_fdr_ < 0.05.

### Data availability

All relevant data without subject identifiers are within the manuscript. Individual imaging data with subject identifiers cannot be shared publicly because of institutional policies regarding data sharing and the protection of research subject privacy. The IRB protocols and the consent under which the subjects received imaging scans did not include language that permits the inclusion of their images in public data repositories. Data are available for researchers who meet the criteria from MGH IRB of MassGeneral Brigham HealthCare. Researchers seeking to utilize the de-identified imaging data from this manuscript should contact B.L.E. and Y.G..B.

## Results

**Figure 2:**
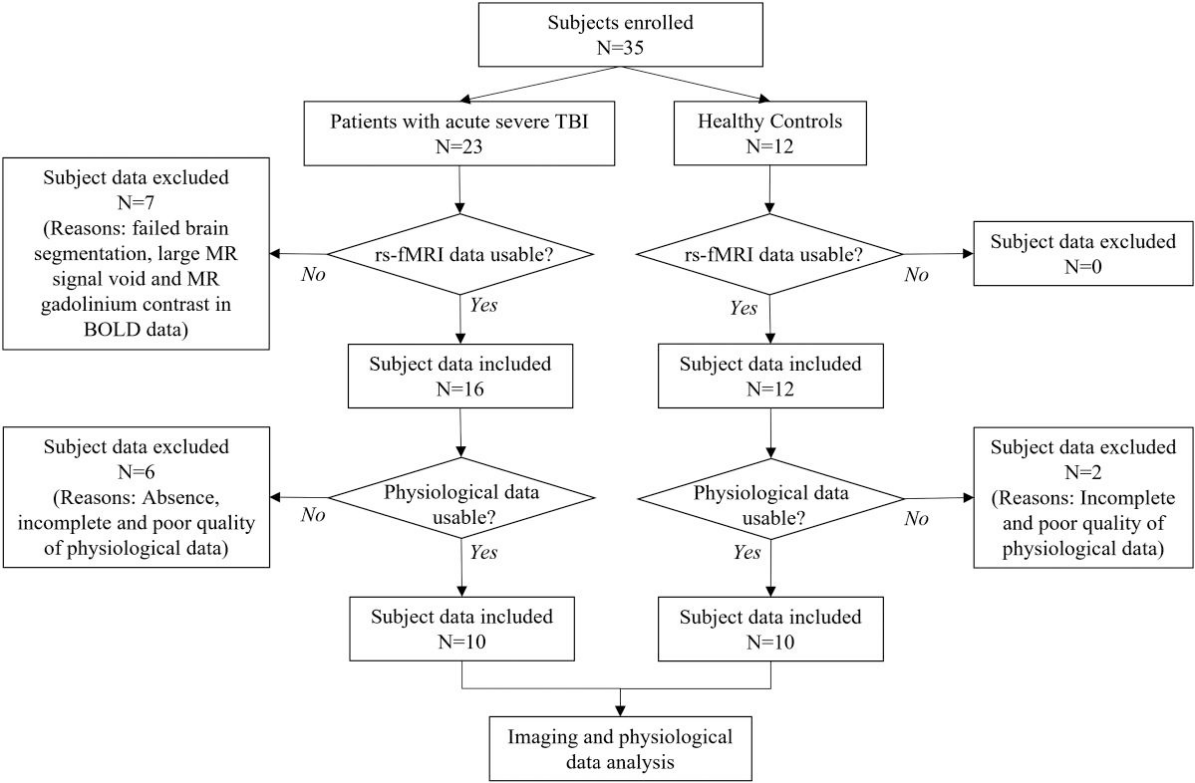
**Subject inclusion and exclusion flow diagram**.

A total of 35 subjects were enrolled (age range 18–78 years). Twenty-three were patients (median = 37.0 years, IQR = 27.5–63.5 years; 15 M), and 12 were healthy controls (median = 32.5 years, IQR = 28.8–35.8 years; 3 M). The schematic diagram of subject inclusion and exclusion is shown in Fig. 2. The head impact mechanism and the level of consciousness at the time of the rs-fMRI scan for patients are shown in Table 1. The distribution of brain lesions in the patients is shown in Supplementary Fig. 2. Lesions in most patients occurred in inferior frontal and anterior temporal areas. Of the 23 enrolled patients, 7 patients were excluded from subsequent data analyses due to failed brain segmentation caused by brain distortion, large MR signal void (e.g. from ventriculoperitoneal shunts), or gadolinium contrast that was administered for clinical imaging before the research MRI sequences (see Table 1 and Fig. 2). No healthy subjects were excluded due to image quality (Table 2).

**Table 1:** Feasibility of physiological monitoring during resting-state functional MRI in critically ill patients

ID	Age	Sex	TBI mechanism	Day of rs-fMRI post-TBI	Usable rs-fMRI data	Presence of plethysmography time series	Presence of respiratory time series	Noise in plethysmography time series	Noise in respiratory time series	Reasons to exclude data
**Patients**										
P1	19	F	Ped struck	25	Y	Y	Y	N	N	N/A
P2	37	M	MVC	5	Y	Y	Y	N	N	N/A
P3	61	M	MVC	6	Y	Y	Y	N	N	N/A
P4	29	M	MVC	3	Y	Y	Y	N	Machine noise	Poor quality of physiological data
P5	20	M	Fall	18	Y	N	Y		N	Incomplete physiological data
P6	47	M	Fall	2	N	Y	Y	N	N	Failed brain segmentation
P8	18	M	MVC	3	Y	Y	Y	N	N	N/A
P9	49	M	Fall	5	Y	Y	Y	N	N	N/A
P10	56	F	MVC	4	N	Y	Y	N	N	MR gadolinium contrast in BOLD data
P14	34	M	MVC	9	N	Y	N	Noisy signals due to bad position		MR gadolinium contrast in BOLD data
P15	73	F	Ped struck	11	Y	Y	Y	N	N	N/A
P16	72	M	MVC	9	Y	Y	Y	N	N	N/A
P20	66	F	Ped struck	2	Y	Y	Y	N	N	N/A
P23	78	M	Fall	4	Y	N	N			No physiological data acquisition
P24	26	M	MVC	21	N	Y	Y	N	N	Failed brain segmentation
P26	67	F	Other non-intentional injury (fall off bike)	8	Y	Y	Y	Noisy signals due to motion	N	Poor quality of physiological data
P32	26	M	Fall	9	Y	Y	Y	N	N	N/A
P33	73	M	Fall	9	Y	Y	Y	N	N	N/A
P34	34	M	Other non-intentional injury (skydiving accident)	8	Y	Y	Y	Noisy signals due to bad position	N	Poor quality of physiological data
P36	20	F	MVC	64	N	Y	Y	N	N	Failed brain segmentation
P37	37	F	Ped struck	17	N	Y	Y	Noisy signals	N	Signal void due to shunt in the right hemisphere
P38	56	M	MVC	4	Y	N	N			No physiological data acquisition
P39	34	F	Other non-intentional injury (struck by basketball)	94	N	Y	Y	N	Noisy signals due to motion	Signal void due to shunt in the right hemisphere

Ped struck, Pedestrian struck my motor vehicle; Y, yes; N, no; N/A, not applicable; MVC, motor vehicle collision.

**Table 2:** Feasibility of physiological monitoring during resting-state functional MRI in healthy controls

ID	Age	Sex	Usable rs-fMRI data	Presence of plethysmography time series	Presence of respiratory time series	Noise in plethysmography time series	Noise in respiratory time series	Reasons to exclude data
**Controls**								
C1	33	F	Y	Y	Y	N	N	N/A
C2	32	M	Y	Y	Y	N	N	N/A
C3	48	F	Y	Y	Y	Intermittent loss of signals	N	Poor quality of physiological data
C4	29	M	Y	Y	Y	N	N	N/A
C5	30	F	Y	Y	Y	N	N	N/A
C6	38	F	Y	Y	Y	N	N	N/A
C7	28	F	Y	Y	Y	N	N	N/A
C8	35	F	Y	Y	Y	N	N	N/A
C12	24	F	Y	N	Y	–	N	Incomplete physiological data
C13	28	F	Y	Y	Y	N	N	N/A
C14	38	F	Y	Y	Y	N	N	N/A
C15	35	M	Y	Y	Y	N	N	N/A

Y, yes; N, no; N/A, not applicable.

### Feasibility of acquiring data for prospective physiological correction

Tables 1 and 2 provide details about why subject data were excluded from the group analyses, and a summary is shown in Fig. 2. Of 28 subjects with usable fMRI data (16 patients, 12 healthy controls), neither cardiac nor respiratory data could be acquired in 2 patients because extra time had been used to prepare these 2 patients for MRI scan, and the MRI scan had to be completed within a limited period of time. The time constraint in data acquisition also resulted in incomplete cardiorespiratory data acquisition (i.e. missing either cardiac or respiratory data) in 1 patient and 1 healthy control. The quality of plethysmography data was poor in 3 patients and 1 healthy subject due to subject motion, suboptimal sensor position, and the intermittent loss of signals that magnetic interferences may cause on physiological signal reception in the scanner.

Examples of poor quality plethysmography data are shown in Supplementary Fig. 3. In total, 14% (4/28) of all subjects had incomplete cardiorespiratory data, and another 14% (4/28) of subjects had poor quality cardiorespiratory data. Thus, 63% of patients with intact fMRI data (10/16) and 83% of healthy controls (10/12) had complete and usable cardiac and respiratory data for physiological correction.

Datasets from those 10 patients (median = 55.0 years, IQR = 28.8–70.5 years; 7 M) and 10 healthy controls (median = 32.5 years, IQR = 29.3–35.0 years; 3 M) were included in the subsequent utility analyses. No significant difference in age (*P* = 0.17) or sex (*P* = 0.18) was found between patients and controls.

### Utility of cardiac and respiratory data for physiological correction

#### Comparison of connectivity strength after RETROICOR correction and bandpass filtering of 0.008–0.125 Hz

Brain connectivity of a representative patient and a control subject after RETROICOR correction and bandpass filtering of 0.008–0.125 Hz are shown in Supplementary Fig. 4. There is no noticeable difference in brain connectivity between the two methods of physiological noise correction (RETROICOR versus BANDPASS pipelines) in the patient or control subject by visual inspection.

**Figure 3:**
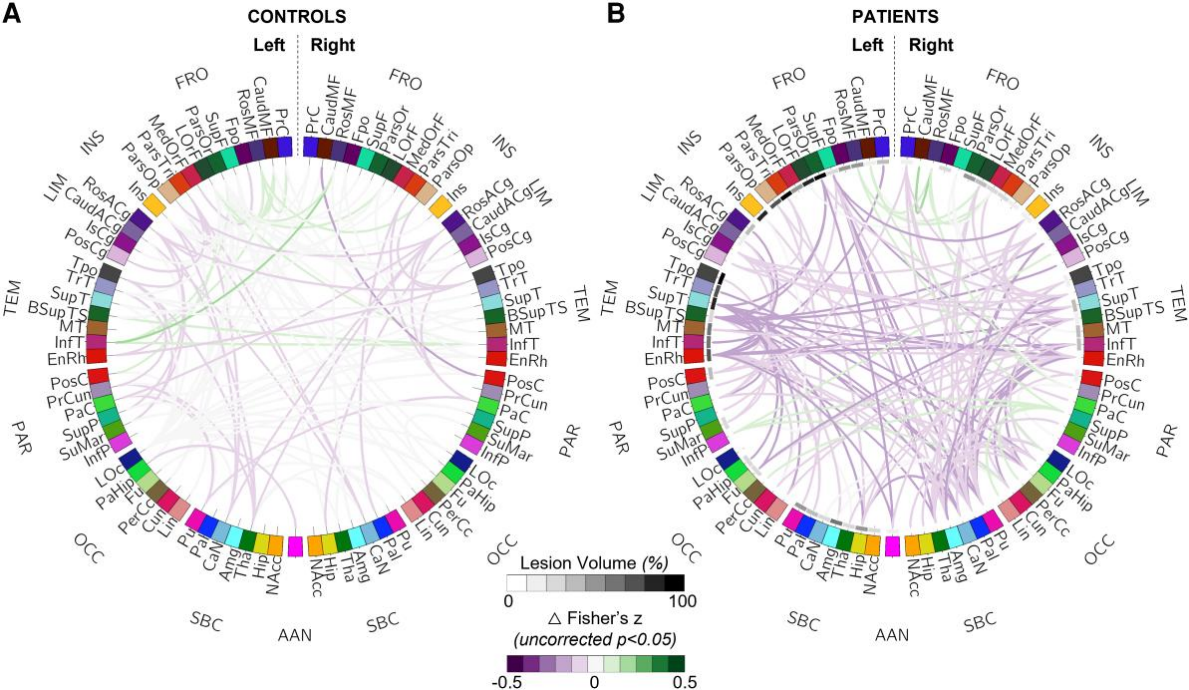
**Group comparisons of brain connectivity between BANDPASS and RETROICOR corrections in controls and patients**. Connectograms showing the comparison of brain connectivity indicated by differences in Fisher-transformed *z*-values between BANDPASS correction of 0.008–0.125 Hz and RETROICOR correction in controls (*n* = 10) (**A**) and patients (*n* = 10) (**B**) before correcting for multiple comparisons. Brain regions were assigned with unique colours defined in FreeSurfer software.^36^ Colour-coded anatomic labels are provided in Table 3. The percentage of voxels in each brain region that contains traumatic lesions is represented by a greyscale ranging from 0 to 100, as shown next to the brain region labels. Purple lines (negative differences in *z*-scores) indicate that functional connectivity between two regions is lower when using the BANDPASS versus RETROICOR pipeline for correcting for cardiorespiratory noise in the rs-fMRI signal. Green lines indicate that functional connectivity between two regions is greater when using the BANDPASS versus RETROICOR pipeline for correcting for cardiorespiratory noise in the rs-fMRI signal. More saturated shades of purple and green colours represent larger differences in brain connectivity between the BANDPASS and RETROICOR pipelines. No significant difference in connectivity between BANDPASS and RETROICOR correction was found after correcting for multiple comparisons (*P*_fdr_ > 0.05).

No significant difference was found in the group comparison of connectivity strength after RETROICOR correction and bandpass filtering of 0.008–0.125 Hz in both patients (Fig. 3A) and healthy subjects (*P*_fdr_ > 0.05) (Fig. 3B).

**Table 3:** Abbreviations of the parcellations used in the connectogram

Abbreviations	Description of the parcellations used in the connectogram
**FRO**	**Frontal**
Fpo	Frontal pole
SupF	Superior frontal
PrC	Precentral
CaudMF	Caudal middle frontal
RosMF	Rostral middle frontal
ParsOp	Pars opercularis
ParsTri	Pars triangularis
ParsOr	Pars orbitalis
**INS**	**Insular**
Ins	Insula
**LIM**	**Limbic**
RosACg	Rostral anterior cingulate
CaudACg	Caudal anterior cingulate
IsCg	Isthmus cingulate
PosCg	Posterior cingulate
**TEM**	**Temporal**
Tpo	Temporal pole
SupT	Superior temporal
BSupTS	Bank of superior temporal sulcus
TrT	Transverse temporal
MT	Middle temporal
InfT	Inferior temporal
EnRh	Entorhinal
**PAR**	**Parietal**
SupP	Superior parietal
InfP	Inferior parietal
SuMar	Supramarginal
PosC	Postcentral
PaC	Paracentral
PrCun	Precuneus
**OCC**	**Occipital**
LOc	Lateral occipital
Cun	Cuneus
PerCc	Pericalcarine
Lin	Lingual
PaHip	Parahippocampal
Fu	Fusiform
**SBC**	**Subcortical**
Pu	Putamen
Pal	Pallidum
CaN	Caudate
NAcc	Accumbens-area
Amg	Amygdala
Tha	Thalamus-proper
Hip	Hippocampus
**AAN**	**Arousal ascending network**

#### Comparison of connectivity strength after RETROICOR correction and bandpass filtering of 15 different frequency bandwidths

**Figure 4:**
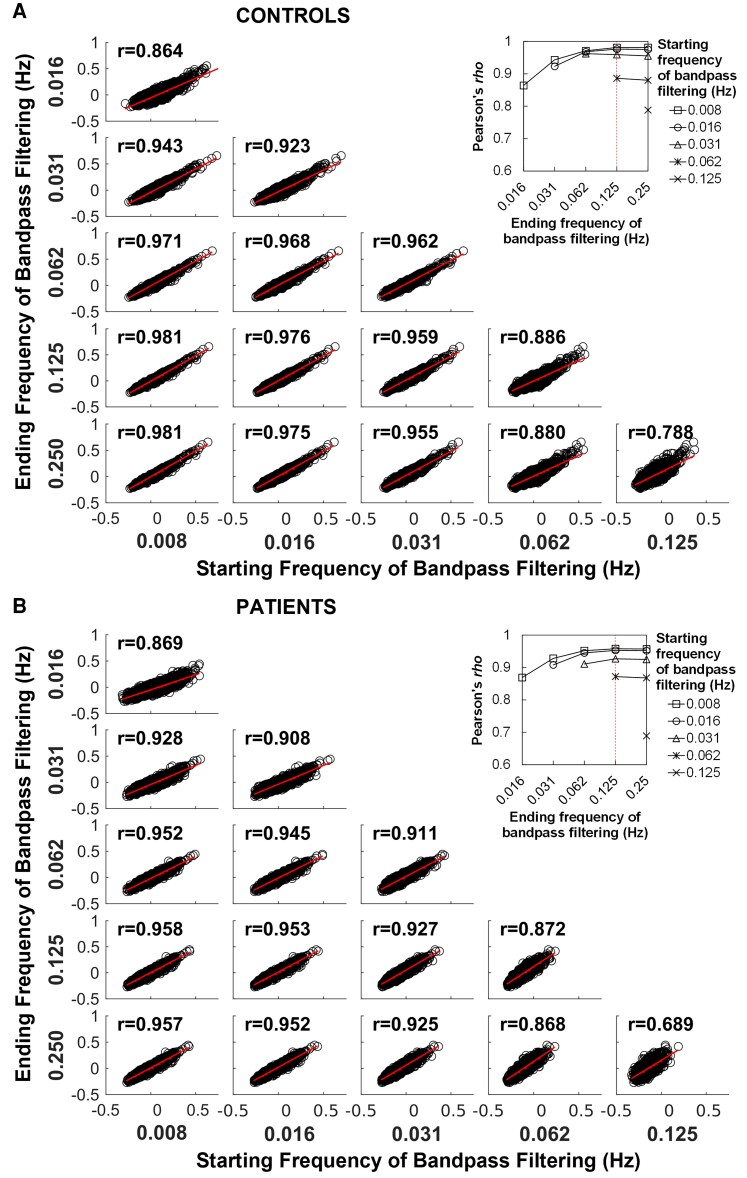
**Correlation of brain connectivity between RETROICOR and BANDPASS pipelines in controls and patients**. Correlation analyses of brain connectivity between RETROICOR and BANDPASS pipelines in controls (*n* = 10) (**A**) and patients (*n* = 10) (**B**). The Pearson’s *r*-values are shown in each correlation analysis (*P* < 0.001). The curves at the right upper corner show the changes of correlation coefficients in the comparisons between the two pipelines. Broken red line indicates the ending frequency of bandpass filtering in BANDPASS pipeline when the highest correlation attains.

The mean Fisher’s *z*-values derived from RETROICOR correction and bandpass filtering in different region pairs started to attain the highest correlation at the bandpass filtering of 0.008–0.125 Hz in patient group (Pearson’s *r* = 0.958, *P* < 0.001) (Fig. 4A) and control group (Pearson’s *r* = 0.981, *P* < 0.001) (Fig. 4B). The correlation stayed relatively high at the bandpass filtering of 0.008–0.25 Hz (patients: Pearson’s *r* = 0.957, *P* < 0.001; controls: Pearson’s *r* = 0.981, *P* < 0.001).

**Figure 5:**
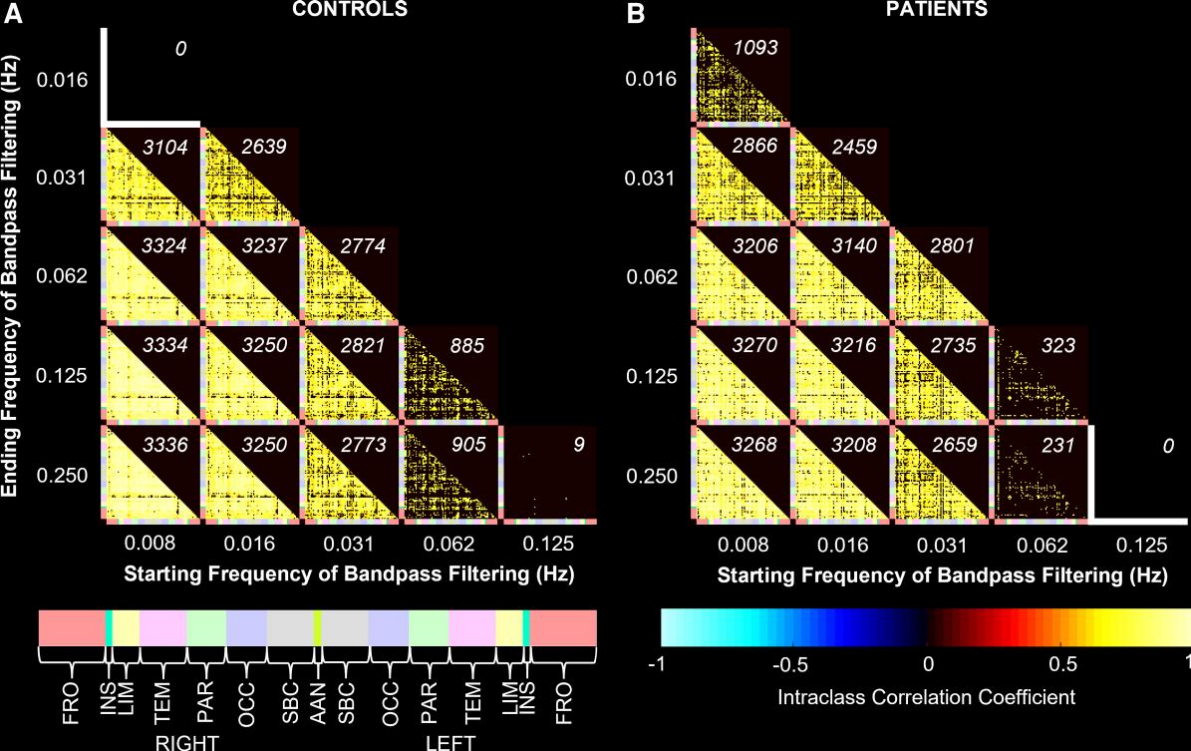
**Intraclass correlation of brain connectivity between RETROICOR and BANDPASS pipelines in controls and patients**. Intraclass correlation of connectivity strength between RETROICOR and BANDPASS pipelines in controls (*n* = 10) (**A**) and patients (*n* = 10) (**B**). The 83 brain regions on the *x*-axis and *y*-axis of each matrix are grouped into cerebral lobes indicated by colours. The number of region pairs showing significant intraclass correlation after correcting for multiple comparisons are shown in *italics* at the upper right hand corner of each matrix. FRO, frontal; INS, insula; LIM, limbic; TEM, temporal; PAR, parietal; OCC, occipital; SBC, subcortical; AAN, arousal ascending network.

An increased number of region pairs had a significant intraclass correlation coefficient between RETROICOR and bandpass filtering with the starting frequency at 0.008 Hz in both patients and controls (Fig. 5). The top two frequency bandwidths with the highest number of region pairs showing significant intraclass correlation are 0.008–0.125 Hz and 0.008–0.25 Hz.

## Discussion

The utility of prospective cardiorespiratory data acquisition for physiological noise correction has been demonstrated in rs-fMRI connectivity studies of healthy subjects^3,15–18,24,25^ and medically stable patients^26,27^ but has not been investigated in rs-fMRI data acquired in critically ill patients. We found that in 6 of 16 critically ill patients with acute severe TBI, we could not conduct prospective cardiorespiratory noise correction due to missing or corrupted physiological data. Furthermore, we found no significant differences in functional brain connectivity for patients or controls when we compared RETROICOR using prospectively collected cardiac and respiratory data versus bandpass filtering at 0.008–0.125 Hz, two independent methods of physiological noise correction that are automated and frequently used for physiological noise correction.^3,15–18,24–27^ Collectively, these findings suggest that prospective cardiorespiratory data acquisition during rs-fMRI has limited feasibility and utility for correcting physiological noise in rs-fMRI data in critically ill patients with acute severe TBI.

### Impact of physiological correction using respiratory and cardiac data in ICU settings

Correction of cardiorespiratory noise that may confound rs-fMRI connectivity data is essential to ensure that BOLD signal modulation and the reduction in the degrees of freedom are identical across subjects.^17^ A common approach to correct for cardiorespiratory noise is to regress prospectively collected cardiac and respiratory signals from the rs-fMRI data using RETROICOR. In our study, the advantage of using individualized cardiorespiratory noise correction via RETROICOR was outweighed by the clinical limitations of acquiring cardiac and respiratory data in critically ill patients. Excellent quality cardiac and respiratory data are required for physiological correction using RETROICOR. However, patients with severe acute TBI often have reduced tolerance for lying supine in the scanner, unstable haemodynamics, peripheral injuries and impaired awareness. Furthermore, patients with critical illness must be scanned on a clinical MRI system that typically lacks precise instrumentation for acquiring cardiac and respiratory data. In our study, these safety considerations and logistical factors made it infeasible to consistently collect the high-quality cardiac and respiratory data required for RETROICOR.

In the absence of prospectively collected cardiac or respiratory data, bandpass filtering, typically at 0.008–0.125 Hz, may be used to correct for physiological noise. Although bandpass filtering is associated with a loss of degrees of freedom due to the removal of frequency bins,^17^ this approach ensures that data can be analysed in a standardized manner across all individuals. We found that brain connectivity metrics derived from RETROICOR and bandpass filtering were similar. We also tested multiple ranges of bandpass filtering and found that brain connectivity corrected with bandpass filtering of 0.008–0.125 Hz had the strongest correlation with brain connectivity corrected with RETROICOR. This bandpass filtering range has been used in many prior studies^3,24,34^ even though it is associated with a loss of degrees of freedom, in our study from 200 to 60.

### Correcting spontaneous fluctuations at 0.125 Hz or higher in critically ill patients

Our study focused on correcting physiological signals due to respiratory^37,38^ and cardiac cycles^39^ with frequencies of 0.15 Hz or higher because physiological fluctuations below 0.125 Hz are related to neuronal signalling, especially in critically ill patients. Fluctuations related to intracranial pressure (0.008–0.03 Hz),^40^ respiratory gas exchange (0.008–0.03 Hz),^21,41^ respiratory variation (∼0.03 Hz),^42,43^ end-tidal carbon dioxide fluctuations (0–0.05 Hz),^44^ variation in arterial pressure (0.05–0.15 Hz),^45,46^ or heart rate variability (0.05–0.15 Hz)^43^ (Supplementary Fig. 5), all occur at a similar frequency to spontaneous BOLD fluctuations. Among these fluctuations, respiratory gas exchange,^21^ respiratory variation,^42^ end-tidal carbon dioxide fluctuations,^44^ and heart rate variability^43^ were previously found to be correlated with oscillations of the default mode network (DMN). Therefore, extra caution is required when removing the physiological ‘noise’ in the frequency bandwidth below 0.125 Hz to avoid removing important information about functional connectivity.

### rs-fMRI in the ICU setting

Resting-state fMRI has historically been used as an investigational tool to study functional brain network connectivity in patients with a broad range of neurological and psychiatric disorders. In critically ill patients with severe brain injuries, emerging evidence suggests that DMN connectivity is associated with recovery of consciousness,^3^ and DMN and salience network connectivity predict long-term functional outcomes.^7^ rs-fMRI is also being used as a pharmacodynamic biomarker in treatment studies aimed at promoting recovery of consciousness.^11^ More recently, rs-fMRI has emerged as a promising clinical tool^47^ for mapping functional brain connectivity and relating it to the capacity for recovery of awareness.^3^ In the early stages of clinical rs-fMRI implementation, it is essential that data acquisition and processing are standardized and that safety and feasibility are optimized. Our findings support this goal and inform future clinical implementation efforts by providing initial evidence that cardiorespiratory data are difficult to collect uniformly and have limited utility for evaluating functional connectivity.

### Limitations of this study

The main limitation of our study is its small sample size, which reflects the difficulty of transporting and scanning critically ill patients in the early days of recovery from severe TBI. A larger sample size may have revealed subtle differences between the RETROICOR and bandpass methods. In addition, we used the plethysmography and chest recordings acquired with the standard physiological monitoring unit in the MRI scanner, which is designed for clinical monitoring and pacing image acquisition for cardiac and abdominal imaging. The physiological monitoring unit was not designed for the quantitative assessment of cardiac and respiratory activity.

Although a larger sample and more precise equipment may have led to different findings, our goal was to assess the feasibility and utility of correcting cardiorespiratory noise in an ICU setting, given the practical challenges of acquiring fMRI data in critically ill patients, particularly patients with acute severe TBI. In this context, our findings indicate that when functional connectivity is integrated into the clinical assessment of critically ill patients, bandpass filtering at 0.008–0.125 Hz can be used as an alternative to prospective acquisition of cardiorespiratory data for physiological correction of the rs-fMRI signal.

## Conclusion

We found that prospective cardiorespiratory correction has limited feasibility and utility in critically ill patients with acute severe TBI. Given currently available technology and the logistical challenges of cardiorespiratory data acquisition on a clinical MRI scanner, our findings suggest that studies using prospective cardiorespiratory correction in critically ill patients may need to exclude a significant number of patients and accept a reduced sample size. We also observed that physiological noise correction using prospective cardiorespiratory data may not provide a significant advantage of analytic utility over retrospective bandpass filtering correction in critically ill patients. These observations suggest that physiological correction of rs-fMRI using prospective acquisition of cardiorespiratory data has limited feasibility and utility in rs-fMRI studies of critically ill patients.

## Supplementary Material

fcac280_Supplementary_Data

## Abbreviations

ΔBOLD =: per cent BOLD signal changes
AAN =: ascending arousal network
AFNI =: Analysis of Functional NeuroImage
BOLD =: blood oxygenation level dependent
DMN =: default mode network
ICU =: intensive care unit
IQR =: interquartile range
RETROICOR =: image-based method for retrospective correction of physiological motion effects in fMRI
rs-fMRI =: resting-state functional MRI
TBI =: traumatic brain injury
TE =: echo time
TR =: repetition time
