# Supplementary material for: Correcting cardiorespiratory noise in resting-state functional MRI data acquired in critically ill patients
Source: Brain Commun. 2022 Oct 31;4(6):fcac280. doi: 10.1093/braincomms/fcac280 (PMC9665273; doi:10.1093/braincomms/fcac280)
Supplement: fcac280_Supplementary_Data [file fcac280_supplementary_data.docx]

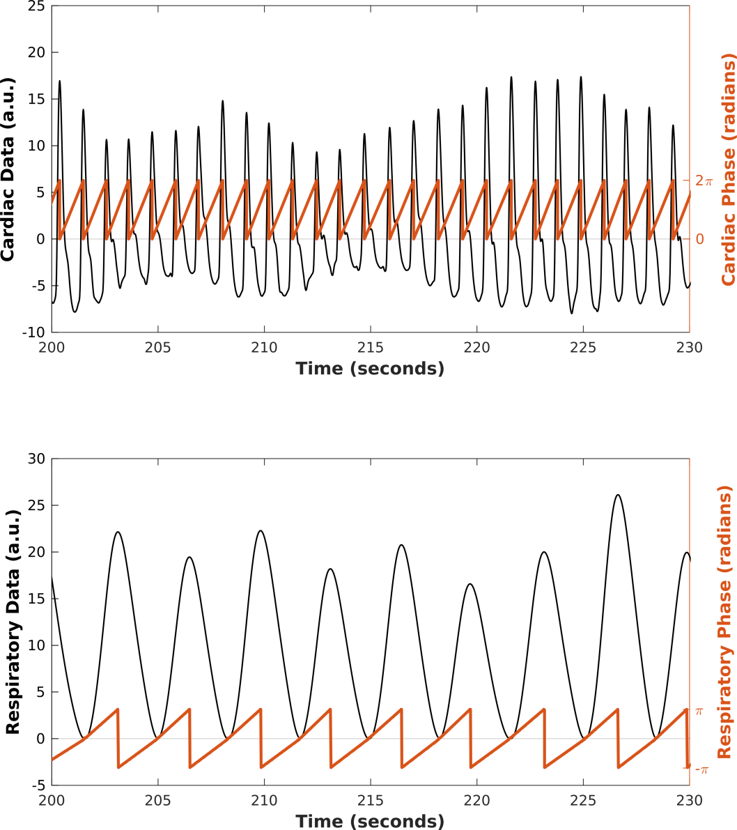


**Supplementary Fig. 1**. **Example of cardiac and respiratory phases derived from cardiac and respiratory data respectively for RETROICOR^1^.** Orange lines indicate the cardiac and respiratory phases used in RETROICOR. The cardiac phase advances linearly from 0 to 2π during each R-R interval and is reset to 0 for the next cycle. The inspiratory phase spans from 0 to π and the expiratory phase spans from 0 to -π.


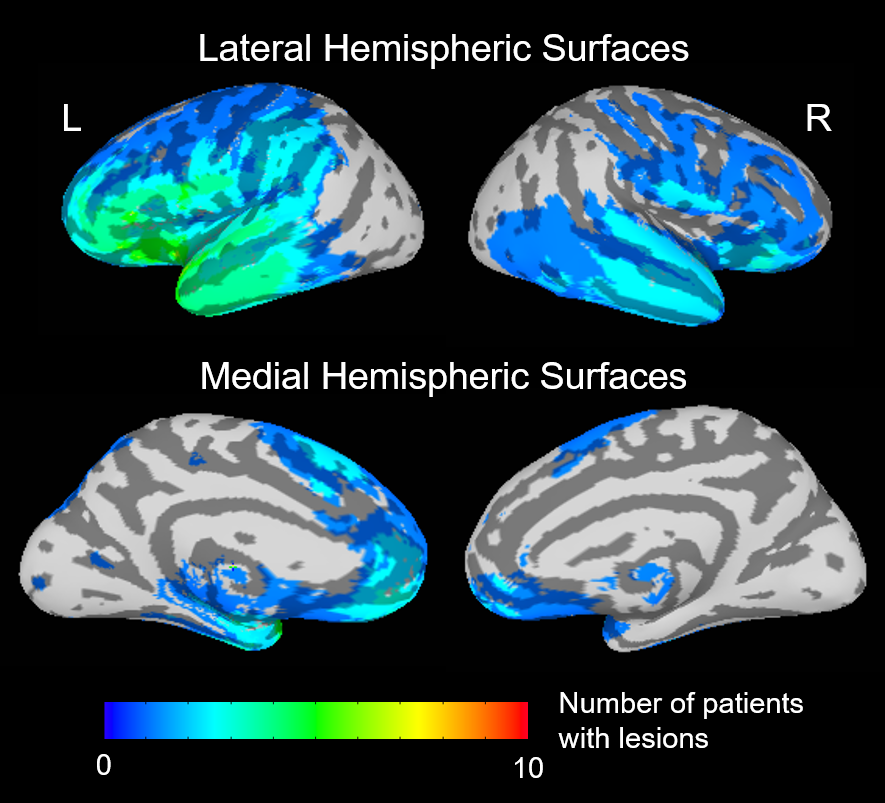


**Supplementary Fig. 2.** **Distribution of brain lesions in the patient group.** Lesions in most patients occurred in inferior frontal and anterior temporal areas (n=10).


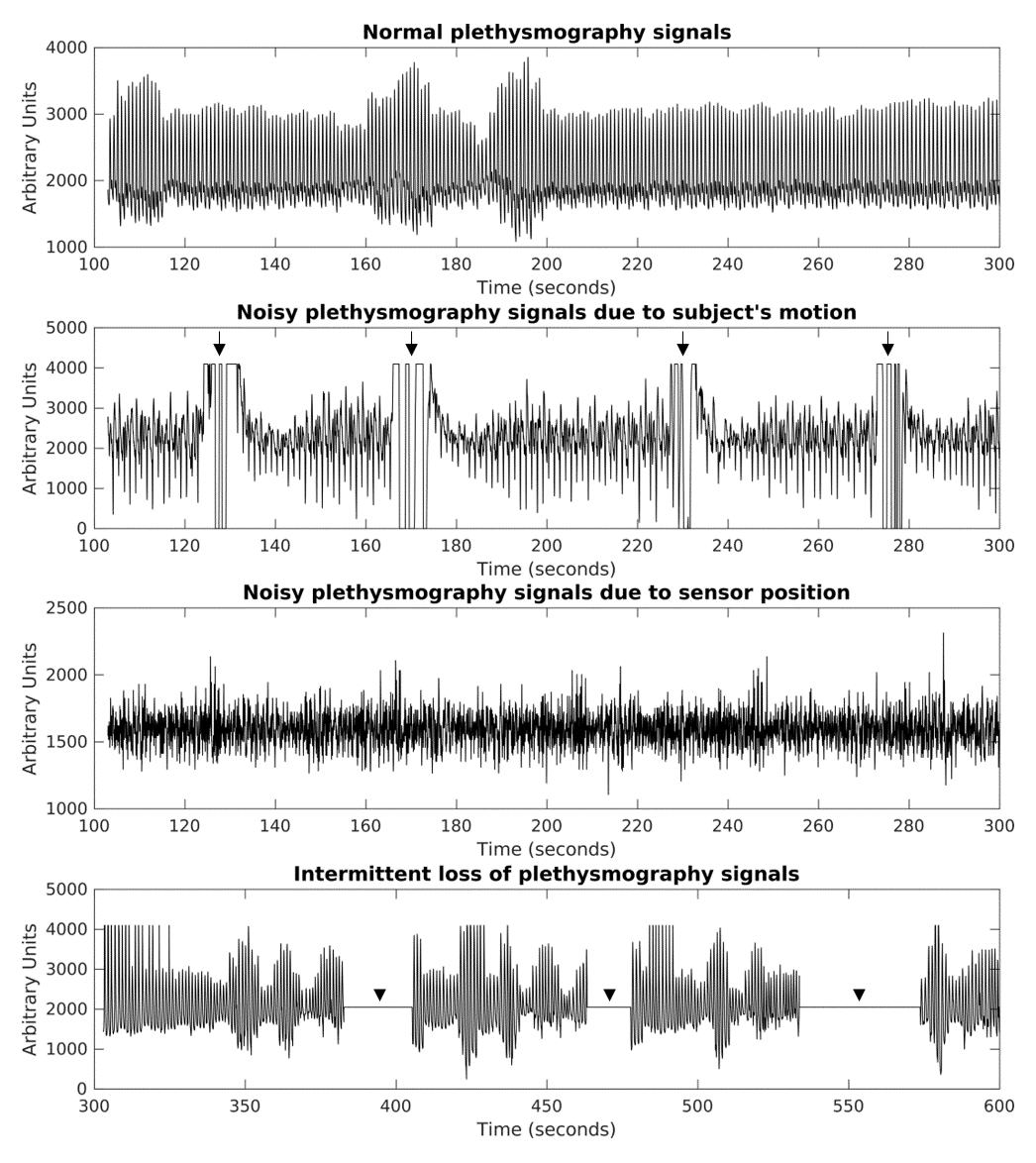


**Supplementary Fig. 3. Examples of normal and poor quality plethysmography signals that could not be used for physiological correction.** Arrows indicate the artifacts due to subject’s motion. Arrowheads indicate the loss of plethysmography signals.

**Supplementary Fig. 4**. **Brain connectivity derived following RETROICOR and BANDPASS pipelines in a representative patient and a control subject.** Connectograms showing the brain connectivity indicated by the Pearson’ correlation coefficients derived following RETROICOR pipeline (upper panel) and bandpass filtering of 0.008-0.125Hz in BANDPASS pipeline (lower panel) in a representative patient and a control subject. The connectivity links shown in the connectograms represent Pearson’s correlation coefficients >0.8 and exist after correcting multiple comparisons (p_fdr_<0.05). Brain regions were assigned with unique colors defined in FreeSurfer software^2^. Color-coded anatomic labels are provided in Table 3. The percentage of voxels in each brain region that contain traumatic lesions is represented by a greyscale ranging from 0 to 100, as shown next to the brain region labels in the patient.

**Supplementary Fig. 5**. **Spontaneous fluctuations at 1Hz or below in resting condition.**  They include intracranial pressure ^3^, respiratory gas exchange ^4,5^, respiratory variation ^6^, end-tidal partial pressure of carbon dioxide ^7^, variation in arterial pressure ^8,9^, and heart rate variability ^10^.

**References**

1. Glover GH, Li TQ, Ress D. Image-based method for retrospective correction of physiological motion effects in fMRI: RETROICOR. *Magn Reson Med*. Jul 2000;44(1):162-7.

2. Destrieux C, Fischl B, Dale A, Halgren E. Automatic parcellation of human cortical gyri and sulci using standard anatomical nomenclature. *Neuroimage*. Oct 15 2010;53(1):1-15. doi:10.1016/j.neuroimage.2010.06.010

3. Lundberg N. Continuous recording and control of ventricular fluid pressure in neurosurgical practice. *Acta Psychiatr Scand Suppl*. 1960;36(149):1-193.

4. Chan ST, Evans KC, Song TY, et al. Dynamic brain-body coupling of breath-by-breath O2-CO2 exchange ratio with resting state cerebral hemodynamic fluctuations. *PLoS One*. 2020;15(9):e0238946. doi:10.1371/journal.pone.0238946

5. Lenfant C. Time-dependent variations of pulmonary gas exchange in normal man at rest. *J Appl Physiol*. Apr 1967;22(4):675-84. doi:10.1152/jappl.1967.22.4.675

6. Birn RM, Diamond JB, Smith MA, Bandettini PA. Separating respiratory-variation-related fluctuations from neuronal-activity-related fluctuations in fMRI. *NeuroImage*. Jul 15 2006;31(4):1536-48. doi:10.1016/j.neuroimage.2006.02.048

7. Wise RG, Ide K, Poulin MJ, Tracey I. Resting fluctuations in arterial carbon dioxide induce significant low frequency variations in BOLD signal. Research Support, Non-U.S. Gov't. *NeuroImage*. Apr 2004;21(4):1652-64. doi:10.1016/j.neuroimage.2003.11.025

8. Mayer S. Studien zur physiologie des herzens und der blutgefasse. V. Ueber spontane blutdruckschwankungen. *Akad Wiss Wien Math Nat Kl 74*. 1876;74:281-307.

9. Obrig H, Neufang M, Wenzel R, et al. Spontaneous low frequency oscillations of cerebral hemodynamics and metabolism in human adults. *Neuroimage*. Dec 2000;12(6):623-39. doi:10.1006/nimg.2000.0657

10. Chang C, Metzger CD, Glover GH, Duyn JH, Heinze HJ, Walter M. Association between heart rate variability and fluctuations in resting-state functional connectivity. *NeuroImage*. Mar 2013;68:93-104. doi:10.1016/j.neuroimage.2012.11.038
